# Prevalence of Health Misinformation on Social Media: Systematic Review

**DOI:** 10.2196/17187

**Published:** 2021-01-20

**Authors:** Victor Suarez-Lledo, Javier Alvarez-Galvez

**Affiliations:** 1 Department of Biomedicine, Biotechnology and Public Health University of Cadiz Cadiz Spain; 2 Computational Social Science DataLab University Research Institute on Social Sciences University of Cadiz Jerez de la Frontera, Cadiz Spain

**Keywords:** social media, health misinformation, infodemiology, infodemics, social networks, poor quality information, social contagion

## Abstract

**Background:**

Although at present there is broad agreement among researchers, health professionals, and policy makers on the need to control and combat health misinformation, the magnitude of this problem is still unknown. Consequently, it is fundamental to discover both the most prevalent health topics and the social media platforms from which these topics are initially framed and subsequently disseminated.

**Objective:**

This systematic review aimed to identify the main health misinformation topics and their prevalence on different social media platforms, focusing on methodological quality and the diverse solutions that are being implemented to address this public health concern.

**Methods:**

We searched PubMed, MEDLINE, Scopus, and Web of Science for articles published in English before March 2019, with a focus on the study of health misinformation in social media. We defined health misinformation as a health-related claim that is based on anecdotal evidence, false, or misleading owing to the lack of existing scientific knowledge. We included (1) articles that focused on health misinformation in social media, including those in which the authors discussed the consequences or purposes of health misinformation and (2) studies that described empirical findings regarding the measurement of health misinformation on these platforms.

**Results:**

A total of 69 studies were identified as eligible, and they covered a wide range of health topics and social media platforms. The topics were articulated around the following six principal categories: vaccines (32%), drugs or smoking (22%), noncommunicable diseases (19%), pandemics (10%), eating disorders (9%), and medical treatments (7%). Studies were mainly based on the following five methodological approaches: social network analysis (28%), evaluating content (26%), evaluating quality (24%), content/text analysis (16%), and sentiment analysis (6%). Health misinformation was most prevalent in studies related to smoking products and drugs such as opioids and marijuana. Posts with misinformation reached 87% in some studies. Health misinformation about vaccines was also very common (43%), with the human papilloma virus vaccine being the most affected. Health misinformation related to diets or pro–eating disorder arguments were moderate in comparison to the aforementioned topics (36%). Studies focused on diseases (ie, noncommunicable diseases and pandemics) also reported moderate misinformation rates (40%), especially in the case of cancer. Finally, the lowest levels of health misinformation were related to medical treatments (30%).

**Conclusions:**

The prevalence of health misinformation was the highest on Twitter and on issues related to smoking products and drugs. However, misinformation on major public health issues, such as vaccines and diseases, was also high. Our study offers a comprehensive characterization of the dominant health misinformation topics and a comprehensive description of their prevalence on different social media platforms, which can guide future studies and help in the development of evidence-based digital policy action plans.

## Introduction

Over the last two decades, internet users have been increasingly using social media to seek and share health information [1]. These social platforms have gained wider participation among health information consumers from all social groups regardless of gender or age [2]. Health professionals and organizations are also using this medium to disseminate health-related knowledge on healthy habits and medical information for disease prevention, as it represents an unprecedented opportunity to increase health literacy, self-efficacy, and treatment adherence among populations [3-9]. However, these public tools have also opened the door to unprecedented social and health risks [10,11]. Although these platforms have demonstrated usefulness for health promotion [7,12], recent studies have suggested that false or misleading health information may spread more easily than scientific knowledge through social media [13,14]. Therefore, it is necessary to understand how health misinformation spreads and how it can affect decision-making and health behaviors [15].

Although the term “health misinformation” is increasingly present in our societies, its definition is becoming increasingly elusive owing to the inherent dynamism of the social media ecosystem and the broad range of health topics [16]. Using a broad term that can include the wide variety of definitions in scientific literature, we here define health misinformation as a health-related claim that is based on anecdotal evidence, false, or misleading owing to the lack of existing scientific knowledge [1]. This general definition would consider, on the one hand, information that is false but not created with the intention of causing harm (ie, misinformation) and, on the other, information that is false or based on reality but deliberately created to harm a particular person, social group, institution, or country (ie, disinformation and malinformation).

The fundamental role of health misinformation on social media has been recently highlighted by the COVID-19 pandemic, as well as the need for quality and veracity of health messages in order to manage the present public health crisis and the subsequent infodemic. In fact, at present, the propagation of health misinformation through social media has become a major public health concern [17]. The lack of control over health information on social media is used as evidence for the current demand to regulate the quality and public availability of online information [18]. In fact, although today there is broad agreement among health professionals and policy makers on the need to control health misinformation, there is still little evidence about the effects that the dissemination of false or misleading health messages through social media could have on public health in the near future. Although recent studies are exploring innovative ways to effectively combat health misinformation online [19-22], additional research is needed to characterize and capture this complex social phenomenon [23].

More specifically, four knowledge gaps have been detected from the field of public health [1]. First, we have to identify the dominant health misinformation trends and specifically assess their prevalence on different social platforms. Second, we need to understand the interactive mechanisms and factors that make it possible to progressively spread health misinformation through social media (eg, vaccination myths, miracle diets, alternative treatments based on anecdotal evidence, and misleading advertisements on health products). Factors, such as the sources of misinformation, structure and dynamics of online communities, idiosyncrasies of social media channels, motivation and profile of people seeking health information, content and framing of health messages, and context in which misinformation is shared, are critical to understanding the dynamics of health misinformation through these platforms. For instance, although the role of social bots in spreading misinformation through social media platforms during political campaigns and election periods is widely recognized, health debates on social media are also affected by social bots [24]. At present, social bots are used to promote certain products in order to increase company profits, as well as to benefit certain ideological positions or contradict health evidence (eg, in the case of vaccines) [25]. Third, a key challenge in epidemiology and public health research is to determine not only the effective impact of these tools in the dissemination of health misinformation but also their impact on the development and reproduction of unhealthy or dangerous behaviors. Finally, regarding health interventions, we need to know which strategies are the best in fighting and reducing the negative impact of health misinformation without reducing the inherent communicative potential to propagate health information with these same tools.

In line with the abovementioned gaps, a recent report represents one of the first steps forward in the comparative study of health misinformation on social media [16]. Through a systematic review of the literature, this study offers a general characterization of the main topics, areas of research, methods, and techniques used for the study of health misinformation. However, despite the commendable effort made to compose a comprehensible image of this highly complex phenomenon, the lack of objective indicators that make it possible to measure the problem of health misinformation is still evident today.

Taking into account this wide set of considerations, this systematic review aimed to specifically address the knowledge gap. In order to guide future studies in this field of knowledge, our objective was to identify and compare the prevalence of health misinformation topics on social media platforms, with specific attention paid to the methodological quality of the studies and the diverse analytical techniques that are being implemented to address this public health concern.

## Methods

### Guideline

This systematic review was conducted according to the Preferred Reporting Items for Systematic Reviews and Meta-Analyses (PRISMA) guidelines [26].

### Inclusion Criteria

Studies were included if (1) the objectives were to address the study of health misinformation on social media, search systematically for health misinformation, and explicitly discuss the impact, consequences, or purposes of misinformation; (2) the results were based on empirical results and the study used quantitative, qualitative, and computational methods; and (3) the research was specifically focused on social media platforms (eg, Twitter, Facebook, Instagram, Flickr, Sina Weibo, VK, YouTube, Reddit, Myspace, Pinterest, and WhatsApp). For comparability, we included studies written in English that were published after 2000 until March 2019.

### Exclusion Criteria

Articles were excluded if they addressed health information quality in general or if they partially mentioned the existence of health misinformation without providing empirical findings. We did not include studies that dealt with content posted on other social media platforms. During the screening process, papers with a lack of methodological quality were also excluded.

### Search Strategy

We searched MEDLINE and PREMEDLINE in March 2019 using the PubMed search engine. Based on previous findings [16], the query searched for MeSH terms and keywords (in the entire body of the manuscript) related to the following three basic analytical dimensions that articulated our research objective: (1) social media, (2) health, and (3) misinformation. The MeSH terms were social media AND health (ie, this term included health behaviors) AND (misinformation OR information seeking behavior OR communication OR health knowledge, attitudes, practice). Based on the results obtained through this initial search, we added some keywords that (having been extracted from the articles that met the inclusion criteria) were specifically focused on the issue of health misinformation on social media. The search using MeSH terms was supplemented with the following keywords: social media (eg, “Twitter” OR “Facebook” OR “Instagram” OR “Flickr” OR “Sina Weibo” OR “YouTube” OR “Pinterest”) AND health AND misinformation (eg, “inaccurate information” OR “poor quality information” OR “misleading information” OR “seeking information” OR “rumor” OR “gossip” OR “hoax” OR “urban legend” OR “myth” OR “fallacy” OR “conspiracy theory”). This initial search retrieved 1693 records. Additionally, this search strategy was adapted for its use in Scopus (3969 records) and Web of Science (1541 records). A full description of the search terms can be found in Multimedia Appendix 1.

### Study Selection

**Figure 1:**
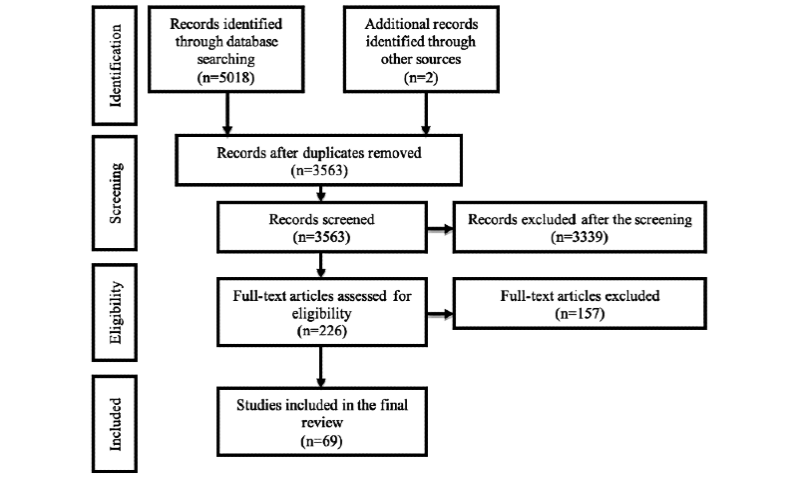
Preferred Reporting Items for Systematic Reviews and Meta-Analyses flow chart.

In total, we collected 5018 research articles. After removing duplicates, we screened 3563 articles and retrieved 226 potentially eligible articles. In the next stage, we independently carried out a full-text selection process for inclusion (k=0.89). Discrepancies were shared and resolved by mutual agreement. Finally, a total of 69 articles were included in this systematic review (Figure 1).

### Data Extraction

In the first phase, the data were extracted by VSL and then checked by VSL and JAG. In order to evaluate the quality of the selected studies and given the wide variety of methodologies and approaches found in the articles, we composed an extraction form based on previous work [27-29]. Each extraction form contained 62 items, most of which were closed questions that could be answered using predefined forms (yes/good, no/poor, partially/fair, etc). Following this coding scheme, we extracted the following four different fields of information: (1) descriptive information (27 items), (2) search strategy evaluation (eight items), (3) information evaluation (six items), and (4) the quality and rigor of methodology and reporting (15 items) for either quantitative or qualitative studies (Multimedia Appendix 1). Questions in field 2, which have been used in previous studies [27], assessed the quality of information provided to demonstrate how well reported, systematic, and comprehensive the search strategy was (S score). The items in field 3 measured how rigorous the evaluation was (E score) for health-related misinformation [27]. Field 4 contained items designed for the general evaluation of quality in the research process, whether quantitative [28] or qualitative [29]. This Q-score approach takes into account general aspects of the research and reporting, such as the study, methodology, and quality of the discussion. For each of the information fields, we calculated the raw score as the sum of each of the items by equating “yes” or “good” as 1 point, “fair” as 0.5 points, and “no” or “poor” as 0 points (Multimedia Appendix 2). The purpose of these questions is to guarantee the quality of the selected studies.

Furthermore, in order to be able to compare the methods used in the selected studies, the studies were classified into several categories. The studies classified as “content/text analysis” used methods related to textual and content analysis, emphasizing the word/topic frequency, linguistic inquiry word count, n-grams, etc. The second category “evaluating content” grouped together studies whose methods were focused on the evaluation of content and information. In general, these studies analyzed different dimensions of the information published on social media. The third category “evaluating quality” included studies that analyzed the quality of the information offered in a global way. This category considered other dimensions in addition to content, such as readability, accuracy, usefulness, and sources of information. The fourth category “sentiment analysis” included studies whose methods were focused on sentiment analysis techniques (ie, methods measuring the reactions and the general tone of the conversation on social media). Finally, the “social network analysis” category included those studies whose methods were based on social network analysis techniques. These studies focused on measuring how misinformation spreads on social media, the relationship between the quality of information and its popularity on these social platforms, the relationship between users and opinions, echochambers effects, and opinion formation.

Of the 226 studies available for full-text review, 157 were excluded for various reasons, including research topics that were not focused on health misinformation (n=133). We also excluded articles whose research was based on websites rather than social media platforms (n=16), studies that did not assess the quality of health information (n=6) or evaluated institutional communication (n=5), nonempirical studies (n=2), and research protocols (n=1). In addition, two papers were excluded because of a lack of quality requirements (Q score <50%). Finally, the protocol of this review was registered at the International Prospective Register of Systematic Reviews (PROSPERO CRD42019136694).

## Results

### Prevalence of Health Misinformation

Ultimately, 69 studies were identified as eligible, and they covered a wide range of health topics and social media platforms, with the most common data source being Twitter (29/69, 43%), followed by YouTube (25/69, 37%) and Facebook (6/69, 9%). The less common sources were Instagram, MySpace, Pinterest, Tumblr, WhatsApp, and VK or a combination of these. Overall, 90% (61/69) of the studies were published in health science journals, and only 7% (5/69) of the studies were published in communication journals. The vast majority of articles analyzed posts written exclusively in one language (63/69, 91%). Only a small percentage assessed posts in more than one language (6/69, 10%).

Table 1 classifies the studies by topic and social media platform [30-97]. It also includes the prevalence of health misinformation posts. The topics were articulated around the following six principal categories: vaccines (22/69, 32%), drugs or smoking issues (16/69, 22%), noncommunicable diseases (13/69, 19%), pandemics (7/69, 10%), eating disorders (6/69, 9%), and medical treatments (5/69, 7%). The quality assessment results for the S score, E score, and Q score are reported in Multimedia Appendix 3.

**Figure 2:**
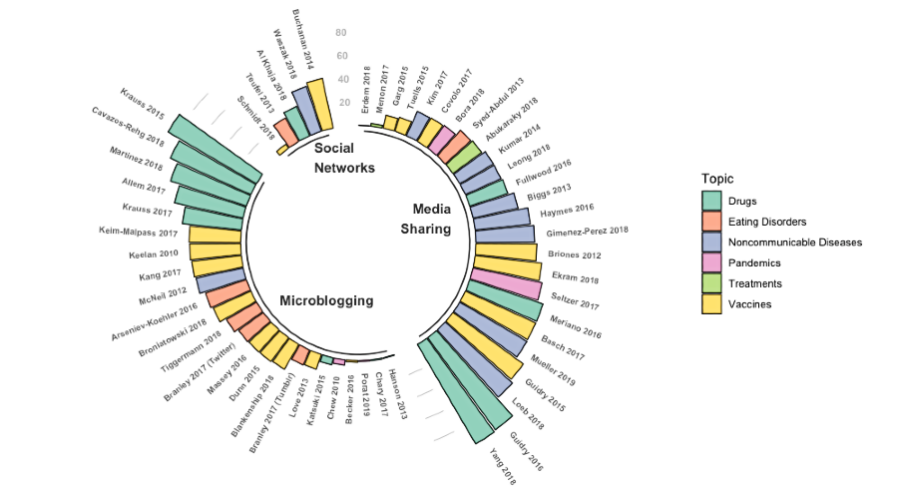
Prevalence of health misinformation grouped by different topics and social media type.

Figure 2 shows the prevalence of health misinformation grouped by different topics and social media typology. Studies are ordered according to the percentage of health misinformation posts found in the studies selected. These works were also classified according to the type of social media under study. In this way, papers focused on Twitter, Tumblr, or Myspace were categorized as “microblogging.” Additionally, papers focused on YouTube, Pinterest, or Instagram were classified within “media sharing” platforms. Moreover, papers focused on Facebook, VK, or WhatsApp were included within the group of “social network” platforms. While all topics were present on all the different social media platforms, we found some differences in their prevalence. On one hand, vaccines, drugs, and pandemics were more prevalent topics on microblogging platforms (ie, Twitter or MySpace). On the other hand, on media sharing platforms (ie, YouTube, Instagram, or Pinterest) and social network platforms (ie, Facebook, VK, or WhatsApp), noncommunicable diseases and treatments were the most prevalent topics. More specifically, Twitter was the most used source for work on vaccines (10/69), drugs or smoking products (10/69), pandemics (4/69), and eating disorders (3/69). For studies on noncommunicable diseases (9/69) or treatments (5/69), YouTube was the most used social media platform.

**Table 1:** Summary of the prevalence of misinformation by topic and social media platform.

Authors	Year	Topic	Social media platform	Prevalence of health misinformation posts
Abukaraky et al [30]	2018	Treatments	YouTube	30%
Ahmed et al [31]	2019	Pandemics	Twitter	N/A^a^
Al Khaja et al [32]	2018	Drugs	WhatsApp	27%
Allem et al [33]	2017	Drugs	Twitter	59%
Allem et al [34]	2017	Drugs	Twitter	N/A
Arseniev-Koehler et al [35]	2016	EDs^b^	Twitter	36%
Basch et al [36]	2017	Vaccines	YouTube	65%
Becker et al [37]	2016	Vaccines	Twitter	1%
Biggs et al [38]	2013	NCDs^c^	YouTube	39%
Blankenship et al [39]	2018	Vaccines	Twitter	24%
Bora et al [40]	2018	Pandemics	YouTube	23%
Branley et al [41]	2017	EDs	Twitter and Tumblr	25%
Briones et al [42]	2012	Vaccines	YouTube	51%
Broniatowski et al [23]	2018	Vaccines	Twitter	35%
Buchanan et al [43]	2014	Vaccines	Facebook	43%
Butler et al [44]	2013	Treatments	YouTube	N/A
Cavazos-Rehg et al [45]	2018	Drugs	Twitter	75%
Chary et al [46]	2017	Drugs	Twitter	0%
Chew et al [47]	2010	Pandemics	Twitter	4%
Covolo et al [48]	2017	Vaccines	YouTube	23%
Dunn et al [49]	2015	Vaccines	Twitter	25%
Dunn et al [50]	2017	Vaccines	Twitter	N/A
Ekram et al [51]	2018	Vaccines	YouTube	57%
Erdem et al [52]	2018	Treatments	YouTube	0%
Faasse et al [53]	2016	Vaccines	Facebook	N/A
Fullwood et al [54]	2016	Drugs	YouTube	34%
Garg et al [55]	2015	Vaccines	YouTube	11%
Gimenez-Perez et al [56]	2018	NCDs	YouTube	50%
Goobie et al [57]	2019	NCDs	YouTube	N/A
Guidry et al [58]	2017	Pandemics	Twitter and Instagram	N/A
Guidry et al [59]	2016	Drugs	Pinterest	97%
Guidry et al [60]	2015	Vaccines	Pinterest	74%
Hanson et al [61]	2013	Drugs	Twitter	0%
Harris et al [62]	2018	EDs	Twitter	N/A
Haymes et al [63]	2016	NCDs	YouTube	47%
Helmi et al [64]	2018	NCDs	Different sources	N/A
Kang et al [65]	2017	Vaccines	Twitter	42%
Katsuki et al [66]	2015	Drugs	Twitter	6%
Keelan et al [67]	2010	Vaccines	MySpace	43%
Keim-Malpass et al [68]	2017	Vaccines	Twitter	43%
Kim et al [69]	2017	NCDs	YouTube	22%
Krauss et al [70]	2017	Drugs	Twitter	50%
Krauss et al [71]	2015	Drugs	Twitter	87%
Kumar et al [72]	2014	NCDs	YouTube	33%
Laestadius et al [73]	2016	Drugs	Instagram	N/A
Leong et al [74]	2018	NCDs	YouTube	33%
Lewis et al [75]	2015	Treatments	YouTube	N/A
Loeb et al [76]	2018	NCDs	YouTube	77%
Love et al [77]	2013	Vaccines	Twitter	13%
Martinez et al [78]	2018	Drugs	Twitter	67%
Massey et al [79]	2016	Vaccines	Twitter	25%
McNeil et al [80]	2012	NCDs	Twitter	41%
Menon et al [81]	2017	Treatments	YouTube	2%
Merianos et al [82]	2016	Drugs	YouTube	65%
Meylakhs et al [83]	2014	NCDs	VK	N/A
Morin et al [84]	2018	Pandemics	Twitter	N/A
Mueller et al [85]	2019	NCDs	YouTube	66%
Porat et al [86]	2019	Pandemics	Twitter	0%
Radzikowski et al [87]	2016	Vaccines	Twitter	N/A
Schmidt et al [88]	2018	Vaccines	Facebook	4%
Seltzer et al [89]	2017	Pandemics	Instagram	60%
Seymour et al [90]	2015	NCDs	Facebook	N/A
Syed-Abdul et al [91]	2013	EDs	YouTube	29%
Teufel et al [92]	2013	EDs	Facebook	22%
Tiggermann et al [93]	2018	EDs	Twitter	29%
Tuells et al [94]	2015	Vaccines	YouTube	12%
van der Tempel et al [95]	2016	Drugs	Twitter	N/A
Waszak et al [96]	2018	NCDs	Facebook	40%
Yang et al [97]	2018	Drugs	YouTube	98%

^a^N/A: not applicable.

^b^EDs: eating disorders.

^c^NCDs: noncommunicable diseases.

Overall, health misinformation was most prevalent in studies related to smoking products, such as hookah and water pipes [33,59,71], e-cigarettes, and drugs, such as opioids and marijuana [45,70,97]. Health misinformation about vaccines was also very common. However, studies reported different levels of health misinformation depending on the type of vaccine studied, with the human papilloma virus (HPV) vaccine being the most affected [67,68]. Health misinformation related to diets or pro–eating disorder arguments were moderate in comparison to the aforementioned topics [35,93]. Studies focused on diseases (ie, noncommunicable diseases and pandemics) also reported moderate misinformation rates [56,85], especially in the case of cancer [76,96]. Finally, the lowest levels of health misinformation were observed in studies evaluating the presence of health misinformation regarding medical treatments. Although first-aid information on burns or information on dental implants was limited in quantity and quality, the prevalence of misinformation for these topics was low. Surgical treatment misinformation was the least prevalent. This was due to the fact that the content related to surgical treatments mainly came from official accounts, which made the online information complete and reliable.

**Figure 3:**
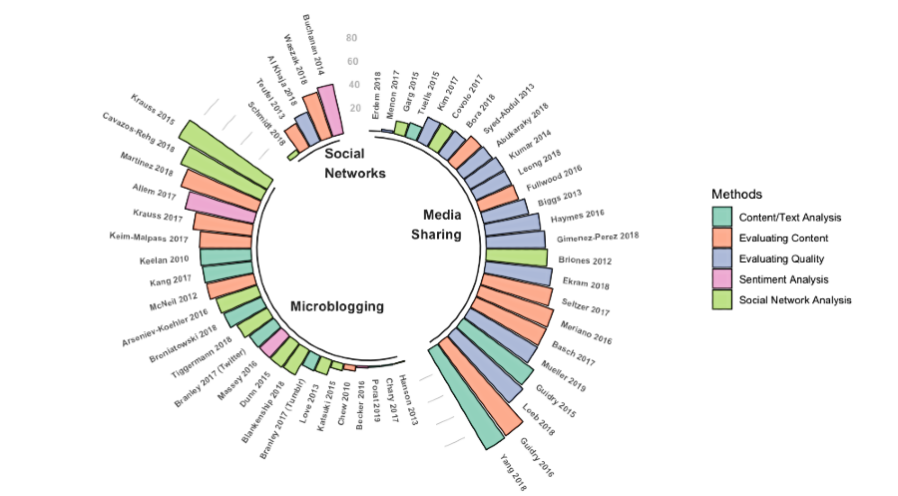
Prevalence of health misinformation grouped by methods and social media type.

Regarding the methods used in the different studies, there were some differences between the diverse social media platforms. We classified the studies based on the methods applied into the following five categories: social network analysis (19/69), evaluating content (18/69), evaluating quality (16/69), content/text analysis (12/69), and sentiment analysis (4/69). Figure 3 shows the different methods applied in the studies classified by the type of social media platform and ordered by the percentage of misinformation posts. Among platforms, such as YouTube and Instagram, methods focused on the evaluation of health information quality and content were common, representing 22% (15/69) and 12% (8/69), respectively. On microblogging platforms, such as Twitter and Tumblr, social network analysis was the method most used by 19% (13/69) of the studies. Finally, on social media platforms, such as Facebook, VK, and WhatsApp, studies whose methods were related to social network analysis represented 3% (2/69) of the included studies and those focused on the evaluation of content represented 4% (3/69) of the included studies.

### Misinformation Topics and Methods

#### Vaccines

Overall, 32% (22/69) of the studies focused on vaccines or vaccination decision-making–related topics. Additionally, 14% (10/69) of the selected articles focused on social media discussion regarding the potential side effects of vaccination [23,36,48,53,55,60,65,77,87,88], 12% (8/69) were centered on the debate around the HPV vaccine [42,49-51,67,68,79,94], and 3% (2/69) were centered on the antivaccine movement [39,43]. According to social media platforms, 9% (6/69) of the studies were focused on the debate and narratives about vaccines in general on Twitter, and 6% (4/69) specifically analyzed the HPV debate on this platform. Papers focused on YouTube also followed a similar trend, and they were centered on the HPV debate and on the public discussion on vaccine side effects and risks for specific population groups (eg, autism in children). Regarding Facebook, all studies were particularly focused on vaccination decision-making.

Most authors studied differences in language use, the effect of a heterogeneous community structure in the propagation of health misinformation, and the role played by fake profiles or bots in the spread of poor quality, doubtful, or ambiguous health content. In line with these concerns, authors pointed out the need to further study the circumstances surrounding those who adopt these arguments [49], and whether alternative strategies to education could improve the fight against antivaccine content [51]. Authors also recommended paying close attention to social media as these tools are assumed to play a fundamental role in the propagation of misinformation. For instance, the role played by the echochamber or the heterogeneous community structure on Twitter has been shown to skew the information to which users are exposed in relation to HPV vaccines [49]. In this sense, it is widely acknowledged that health professionals should pay more attention to antivaccine arguments on social media, so that they can better respond to patients’ concerns [36,43,65,77]. Furthermore, governmental organizations could also use social media platforms to reach a greater number of people [39,55].

#### Drugs and Smoking

Several studies (16/69, 22%) covered misuse and misinformation about e-cigarettes, marijuana, opioid consumption, and prescription drug abuse. Studies covering the promotion of e-cigarette use and other forms of smoking, such as hookah (ie, water pipes or narghiles) represented 7% (5/69) of the articles analyzed. The rest (16%, 11/69) were focused on the analysis of drug misinformation.

According to topic, regarding drug and opioid use, studies investigated the dissemination of misinformation through social media platforms [32,45,46,70,97], the consumption of misinformation related to these products, drug abuse, and the sale of online medical products [61,66]. These studies highlighted the risk, especially for young people, caused by the high rate of misinformation related to the dissemination of drug practice and misuse (predominantly marijuana and opioids) [45]. In addition, social media platforms were identified as a potential source of illegal promotion of the sale of controlled substances directly to consumers [66]. Most drug-related messages on social media were potentially misleading or false claims that lacked credible evidence to support them [32]. Other studies pointed to social media as a potential source of information that illegally promotes the sale of controlled prescription drugs directly to consumers [66]. In the case of cannabinoids, there was often content that described, encouraged, promoted [54], or even normalized the consumption of illicit substances [70].

Unlike drug studies, most of the papers analyzed how e-cigarettes and hookah [33,34,59,71,73,78,82,95] are portrayed on social media and/or the role of bots in promoting e-cigarettes. Regarding e-cigarettes, studies pointed out the high prevalence of misinformation denying health damage [95]. In this sense, it is worth noting the importance of sources of misinformation. While in the case of vaccines, the source of health misinformation was mainly individuals or groups of people with a particular interest (eg, antivaccine movement), social media was found to be frequently contaminated by misinformation from bots (ie, software applications that autonomously run tasks such as spreading positive discourse about e-cigarettes and other tobacco products) [78]. In fact, these fake accounts may influence the online conversation in favor of e-cigarettes given the scientific appearance of profiles [78]. Some of the claims found in this study denied the harmfulness of e-cigarettes. In line with these findings, other studies pointed to the high percentage of messages favoring e-cigarettes as an aid to quitting smoking [95].

We found that 10% (7/69) of the studies used methods focused on evaluating the content of the posts. These studies aimed to explore the misperceptions of drug abuse or alternative forms of tobacco consumption. Along these lines, another study (1/69, 1%) focused on evaluating the quality of content. The authors evaluated the truthfulness of claims about drugs. In particular, we found that 7% (5/69) of the studies used social network analysis techniques. These studies analyzed the popularity of messages based on whether they promoted illegal access to drugs online and the interaction of users with this content. Other studies (3/69, 3%) used content analysis techniques. These studies evaluated the prevalence of misinformation on platforms and geographically, as a kind of “toxicosurveillance” system [34,46].

#### Noncommunicable Diseases

A relevant proportion (13/69, 19%) of studies assessed noncommunicable diseases, such as cancer, diabetes, and epilepsy. Most of the studies focused on the objective evaluation of information quality on YouTube [38,56,57,69,72,74,76,80,85]. Overall, 13% (9/69) of these studies used methods to assess the quality of the information. The authors analyzed the usefulness and accuracy of the information. Moreover, 4% (3/69) of the studies used methods related to content assessment. The main objective of these studies was to analyze which are the most common misinformation topics. Furthermore, 3% (2/69) used social network analysis, and the main objective of the analysis was to study the information dissemination patterns or the social spread of scientifically inaccurate health information.

Some studies evaluated the potential of this platform as a source of information specially for health students or self-directed education among the general public. Unfortunately, the general tone of research findings was that YouTube is not an advisable source for health professionals or health information seekers. Regarding diabetes, the probability of finding misleading videos was high [56]. Misleading videos promoted cures for diabetes, negated scientific arguments, or provided treatments with no scientific basis. Furthermore, misleading videos related to diabetes were found to be more popular than those with evidence-based health information [74], which increased the probability of consuming low-quality health content. The same misinformation pattern was detected for other chronic diseases such as hypertension [72], prostate cancer [76], and epilepsy [80].

#### Pandemics and Communicable Diseases

Results indicated that 10% (7/69) of the studies covered misinformation related to pandemics and communicable diseases such as H1N1 [31,47], Zika [40,89], Ebola [58,84], and diphtheria [86]. All these studies analyzed how online platforms were used by both health information seekers and health and governmental authorities during the pandemic period.

We found that 14% (10/69) of the studies on this topic evaluated the quality of the information. To achieve this, most of the studies used external instruments such as DISCERN and AAD7 Self-Care Behaviors. Overall, 9% (6/69) of the papers evaluated the content of the information. These studies were focused on analysis of the issues of misinformation. Another 4% (3/69) used social media analysis to observe the propagation of misinformation. Finally, 3% (2/69) used textual analysis as the main method. These studies focused on the study of the prevalence of health misinformation.

These studies identified social media as a public forum for free discussion and indicated that this freedom might lead to rumors on anecdotal evidence and misunderstandings regarding pandemics. Consequently, although social media was described as a forum for sharing health-related knowledge, these tools are also recognized by researchers and health professionals as a source of misinformation that needs to be controlled by health experts [83,84]. Therefore, while social media serves as a place where people commonly share their experiences and concerns, these platforms can be potentially used by health professionals to fight against false beliefs on communicable diseases (eg, as it is happening today during the COVID-19 pandemic). Accordingly, social media platforms have been found to be powerful tools for health promotion among governmental institutions and health-related workers, and new instruments that, for instance, are being used to increase health surveillance and intervention against false beliefs and misinformation [31,89]. In fact, different authors agreed that governmental/health institutions should increase their presence on social media platforms during pandemic crises [47,58,84,86].

#### Diet/Eating Disorders

Studies focusing on diet and eating disorders represented 9% (6/69) of the included studies. This set of studies identified pro–eating disorder groups and discourses within social media [35], and how pro–eating disorder information was shared and spread on these platforms [91]. Anorexia was the most studied eating disorder along with bulimia. Furthermore, discourses promoting fitness or recovery after an eating disorder were often compared with those issued by pro–eating disorder groups [41,62,92,93]. In general, the authors agreed on the relevance of pro–eating disorder online groups, the mutual support among members, and the way they reinforce their opinions and health behaviors [35].

Overall, 4% (3/69) of the studies used social network analysis techniques. The authors focused on analyzing the existing connections between individuals in the pro–eating disorder community and their engagement, or comparing the cohesion of these communities with other communities, such as the fitness community, that promote healthier habits. Moreover, 3% (2/69) of the studies evaluated the quality of the content and particularly focused on informative analysis of the videos, that is, the content was classified as informative when it described the health consequences of anorexia or proana if, on the contrary, anorexia was presented as a fashion or a source of beauty. Furthermore, only one study used content analysis techniques. The authors classified the posts according to the following categories: proana, antiana, and prorecovery. Pro–eating disorder pages tended to identify themselves with body-associated pictures owing to the importance they attributed to motivational aspects of pro–eating disorder communities [92]. The pro–eating disorder claims contained practices about weight loss, wanting a certain body type or characteristic of a body part, eating disorders, binge eating, and purging [62]. Pro–eating disorder conversations also had a high content of social support in the form of tips and tricks (eg, “Crunch on some ice chips if you are feeling a hunger craving. This will help you feel as if you are eating something substantial” and “How do you all feel about laxatives?”) [92].

Regarding eating disorders on social media, paying attention to community structure is important according to authors. Although it is widely acknowledged that communities can be positive by providing social support, such as recovery and well-being, certain groups on social media may also reaffirm the pro–eating disorder identity [35]. In fact, polarized pro/anti–eating disorder communities can become closed echochambers where community members are selectively exposed to the content they are looking for and therefore only hear the arguments they want to hear. In this case, the echochamber effect might explain why information campaigns are limited in scope and often encourage polarization of opinion, and can even reinforce existing divides in pro–eating disorder opinions [88].

#### Treatments and Medical Interventions

Finally, we found that 7% (5/69) of the studies assessed the quality of health information regarding different medical treatments or therapies recommended through social media [63,81]. According to method, 6% (4/69) of the studies evaluated the quality of information related to the proposed treatments and therapies. In this sense, the fundamental goal of these studies was aimed at assessing the quality and accuracy of the information.

As in the case of noncommunicable diseases, professionals scanned social networks, especially YouTube, and evaluated the quality of online health content as an adequate instrument for self-care or for health student training. There were specific cases where information was particularly limited in quality and quantity, such as dental implants and first-aid information on burns [30,44]. However, most surgical treatments or tools were found to have a sufficient level of quality information on YouTube [52,81]. In relation to this topic, it is worth pointing out the source of the misinformation. In this particular case, most of the posts were published by private companies. They used the platforms to promote their medical products. Therefore, the amount of misinformation was considerably low compared with other topics, such as eating disorders and vaccines, that are closely linked to the general public. In general, the videos were accurate, were well presented, and framed treatments in a useful way for both health workers and health information seekers.

A full description of the objectives and main conclusions of the reviewed articles is presented in Multimedia Appendix 4.

## Discussion

### Main Findings

This work represents, to our knowledge, the first effort aimed at finding objective and comparable measures to quantify the extent of health misinformation in the social media ecosystem. Our study offers an initial characterization of dominant health misinformation topics and specifically assesses their prevalence on different social platforms. Therefore, our systematic review provides new insights on the following unanswered question that has been recurrently highlighted in studies of health misinformation on social media: How prevalent is health misinformation for different topics on different social platform types (ie, microblogging, media sharing, and social networks)?

We found that health misinformation on social media is generally linked to the following six topical domains: (1) vaccines, (2) diets and eating disorders, (3) drugs and new tobacco products, (4) pandemics and communicable diseases, (5) noncommunicable diseases, and (6) medical treatments and health interventions.

With regard to vaccines, we found some interesting results throughout the different studies. Although antivaccine ideas have been traditionally linked to emotional discourse against the rationality of the scientific and expert community, we curiously observed that in certain online discussions, antivaccine groups tend to incorporate scientific language in their own discourse with logically structured statements and/or with less usage of emotional expressions [53]. Thus, the assimilation of the scientific presentation and its combination with anecdotal evidence can rapidly spread along these platforms through a progressive increment of visits and “likes” that can make antivaccine arguments particularly convincing for health information seekers [53,55]. Furthermore, we found that the complex and heterogeneous community structure of these online groups must be taken into account. For instance, those more exposed to antivaccine information tend to spread more negative concerns about vaccines (ie, misinformation or opinions related to vaccine hesitancy) than users exposed to positive or neutral opinions [49]. Therefore, negative/positive opinions are reinforced through the network structure of particular social media platforms. Moreover, fake profiles tend to amplify the debate and discussion, thereby undermining the possible public consensus on the effectiveness and safety of vaccines, especially in the case of HPV; measles, mumps, and rubella (MMR); and influenza [23].

As observed in our review, health topics were omnipresent over all social media platforms included in our study; however, the health misinformation prevalence for each topic varied depending on platform characteristics. Therefore, the potential effect on population health was ambivalent, that is, we found both positive and negative effects depending on the topic and on the group of health information seekers. For instance, content related to eating disorders was frequently hidden or not so evident to the general public, since pro–eating disorder communities use their own codes to reach specific audiences (eg, younger groups) [98]. To provide a simple example, it is worth mentioning the usage of nicknames, such as proana for proanorexia and promia for probulimia, as a way to reach people with these health conditions and make it easier for people to talk openly about their eating disorders. More positively, these tools have been useful in prevention campaigns during health crises. For example, during the H1N1, Ebola, and Zika pandemics, and, even more recently, with the ongoing COVID-19 pandemic, platforms, such as Twitter, have been valuable instruments for spreading evidence-based health knowledge, expert recommendations, and educative content aimed at avoiding the propagation of rumors, risk behaviors, and diseases [31,89].

Throughout our review, we found different types of misinformation claims depending on the topic. Concerning vaccines, misinformation was often framed with a scientific appearance against scientific evidence [53]. Drug-related misinformation promoted the consumption and abuse of these substances [66]. However, these statements lacked scientific evidence to support them [32]. As with vaccines, false accounts that influenced the online conversation did so with a scientific appearance in favor of e-cigarettes [82]. In this sense, most accounts tended to promote the use and abuse of these items. With beauty as the final goal, misinformation about eating disorders promoted changes in the eating habits of social media users [91]. Furthermore, we found that social media facilitated the development of pro–eating disorder online communities [35]. In general, the results indicated that this type of content promoted unhealthy practices while normalizing eating disorders. In contrast, epidemic/pandemic-related misinformation was not directly malicious. Misinformation on this topic involved rumors, misunderstandings, and doubts arising from a lack of scientific knowledge [31]. The statements were within the framework of the health emergency arising from the pandemic. In line with these findings, we noted findings related to noncommunicable diseases. Messages that focused on this topic promoted cures for chronic diseases or for conditions with no cure through fallacies or urban legends [85].

In this study, we focused on analysis of the results obtained and the conclusions of the authors. Some of our findings are in line with those obtained in recent works [16]. The reviewed studies indicate, on one hand, the difficulty in characterizing and evaluating the quality of health information on social media [1] and, on the other, the conceptual fuzziness that can result from the convergence of multiple disciplines trying to apprehend the multidisciplinary and complex phenomenon of health misinformation on social media. This research field is being studied by health and social scientists [70,73], as well as by researchers from the fields of computer science, mathematics, sociophysics, etc [99,100]. Therefore, we must understand that the inherent multidisciplinary and methodological diversity of studies and the highly dynamic world of social media are a perfect match for making it more difficult to identify comprehensive and transversal solutions to the problem of health misinformation. In fact, as we have found, misinformation on vaccines, drugs, and new smoking products is more prevalent on media-sharing platforms (eg, YouTube) and microblogging applications (eg, Twitter), while misinformation on noncommunicable diseases is particularly prevalent on media sharing platforms where users can widely describe disease symptoms, medical treatments, and therapies [76,85]. Platforms, such as YouTube, owing to their characteristics, allow more space for users to share this type of information, while the natural dynamism of Twitter makes it an ideal medium for discussion among online communities with different political or ideological orientations (eg, pro/antivaccination communities).

Finally, we should mention that the current results are limited to the availability and quality of social media data. Although the digitalization of social life offers researchers an unprecedented amount of health and social information that can be used to understand human behaviors and health outcomes, accessing this online data is becoming increasingly difficult, and some measures have to be taken to mitigate bias [40,43,67,79]. Over the last few years, new concerns around privacy have emerged and led governments to tighten regulations around data access and storage [101,102]. Consequently, in response to these new directives, as well as scandals involving data sharing and data breaches such as the Cambridge Analytica case, social media companies are developing new controls and barriers to data in their platforms. This is why free access to application programming interfaces (APIs) is becoming increasingly difficult and the range of social data accessible via APIs is gradually decreasing. These difficulties in accessing data are also determining which platforms are most frequently used by researchers, which are not used, and which will be used in the near future.

### Limitations and Strengths

The present study has some limitations. First, the conceptual definition of health misinformation is one limitation. In any case, taking into account that we were facing a new field of study, we considered a broad definition in order to be more inclusive and operative in the selection of studies. Therefore, we included as many papers as possible for the review in order to perform an analysis of the largest number of possible topics. Second, from a methodological perspective, our findings are limited to research published in English language journals and do not cover all the social media platforms that exist. Besides, we discovered some technical limitations when conducting this systematic review. Owing to the newness of this research topic, our study revealed difficulties in comparing different research studies characterized by specific theoretical approaches, working definitions, methodologies, data collection processes, and analytical techniques. Some studies selected involved observational designs (using survey methods and textual analysis), whereas others were based on the application of automatic or semiautomatic computational procedures with the aim of classifying and analyzing health misinformation on social media. Finally, taking into account the particular features of each type of social media (ie, microblogging service, video sharing service, or social network) and the progressive barriers in accessing social media data, we need to consider the information and selection bias when studying health misinformation on these platforms. According to these biases, we should ponder which users are behind these tools and how we can extrapolate specific findings (ie, applied to certain groups and social media platforms) to a broader social context.

Despite the limitations described above, it is necessary to mention the strengths of our work. First, we believe that this study represents one of the first steps in advancing research involving health misinformation on social media. Unlike previous work, we offer some measures that can serve as guidance and a comparative baseline for subsequent studies. In addition, our study highlights the need to redirect future research toward social media platforms, which, perhaps due to the difficulties of automatic data collection, are currently being neglected by researchers. Our study also highlights the need for both researchers and health professionals to explore the possibility of using these digital tools for health promotion and the need for them to progressively colonize the social media ecosystem with the ultimate goal of combating the waves of health misinformation that recurrently flood our societies.

### Conclusion

Health misinformation was most common on Twitter and on issues related to smoking products and drugs. Although we should be aware of the difficulties inherent in the dynamic magnitude of online opinion flows, our systematic review offers a comprehensive comparative framework that identifies subsequent action areas in the study of health misinformation on social media. Despite the abovementioned limitations, our research presents some advances when compared with previous studies. Our study provides (1) an overview of the prevalence of health misinformation identified on different social media platforms; (2) a methodological characterization of studies focused on health misinformation; and (3) a comprehensive description of the current research lines and knowledge gaps in this research field.

According to the studies reviewed, the greatest challenge lies in the difficulty of characterizing and evaluating the quality of the information on social media. Knowing the prevalence of health misinformation and the methods used for its study, as well as the present knowledge gaps in this field will help us to guide future studies and, specifically, to develop evidence-based digital policy action plans aimed at combating this public health problem through different social media platforms.

## Appendix

Multimedia Appendix 1 Search terms and results from the search query.

Multimedia Appendix 2 Data extraction sheet.

Multimedia Appendix 3 Summary of quality scores.

Multimedia Appendix 4 Summary table with objectives and conclusions about misinformation prevalence in social media.

## Abbreviations

API: application programming interface
HPV: human papilloma virus
