# Supplementary material for: Prevalence of Health Misinformation on Social Media: Systematic Review
Source: J Med Internet Res. 2021 Jan 20;23(1):e17187. doi: 10.2196/17187 (PMC7857950; doi:10.2196/17187)
Supplement: Multimedia Appendix 1 [file jmir_v23i1e17187_app1.docx]

## Multimedia Appendix 1. Search terms and results from the search query.

Search Query:

(((((((((((social media[MeSH Terms]) OR twitter) OR facebook) OR instagram) OR flickr) OR “sina weibo”) OR YouTube) OR reddit) OR pinterest)) AND ((health[MeSH Terms]) OR health)) AND (((((((((((((((((((((((((misinformation[MeSH Terms]) OR information seeking behavior[MeSH Terms]) OR communication[MeSH Terms]) OR health knowledge, attitudes, practice[MeSH Terms]) OR “inaccurate information”) OR “poor quality information”) OR “low quality information”) OR “health misinformation”) OR “misleading information”) OR “seeking information”) OR rumour) OR rumor) OR rumours) OR rumors) OR gossip) OR hoax) OR hoaxes) OR “urban legend”) OR “urban legends”) OR myth) OR myths) OR fallacy) OR fallacies) OR “conspiracy theories”) OR “conspiracy theory”).

Results of the search query in PubMed

| **Blocks** |  | **Searches PubMed** | **Results** |
| --- | --- | --- | --- |
| **Social Media** | 1 | MeSH: Social Media | 5624 |
|  | 2 | Free terms: “twitter” OR “facebook” OR “instagram” OR “flickr” OR "sina weibo" OR “YouTube” OR “reddit” OR “pinterest” | 6070 |
|  | 3 | 1 OR 2 | 9503 |
| **Health** | 4 | MeSH: health | 336566 |
|  | 5 | Free terms: health | 4346920 |
|  | 6 | 4 OR 5 | 4346920 |
| **Misinformation** | 7 | MeSH: misinformation OR information seeking behavior OR communication OR health knowledge, attitudes, practice | 378428 |
|  | 8 | Free terms: “inaccurate information” OR “poor quality information” OR “low quality information” OR “health misinformation” OR “misleading information” OR “seeking information” OR rumour OR rumor OR rumours OR rumors OR gossip OR hoax OR hoaxes OR “urban legend” OR “urban legends” OR myth OR myths OR fallacy OR fallacies OR “conspiracy theories” OR “conspiracy theory” | 23177 |
|  | 9 | 7 OR 8 | 398955 |
| **Results** | 10 | 3 AND 6 AND 9 | 1693 |
