# Supplementary material for: Prevalence of Health Misinformation on Social Media: Systematic Review
Source: J Med Internet Res. 2021 Jan 20;23(1):e17187. doi: 10.2196/17187 (PMC7857950; doi:10.2196/17187)
Supplement: Multimedia Appendix 2 [file jmir_v23i1e17187_app2.docx]

**Multimedia Appendix 2.** Data extraction sheet.

| **Dimension** | **Items** |
| --- | --- |
| **Search Quality (SQ)** | 1. Was search date/period mentioned? |
|  | 2. Was search tools mentioned? |
|  | 3. Was more than 1 search tool used? |
|  | 4. Was search terms mentioned? |
|  | 5. Was user engagement mentioned? |
|  | 6. Was initial hits reported? |
|  | 7. Was posts in more than 1 language assessed? |
|  | 8. Was interrater reliability for post selection determined |
| **Evaluation Quality (EQ)** | 1. Raters blinded for the source |
|  | 2. Number of raters reported |
|  | 3. More than 1 rater |
|  | 4. Interrater reliability figure for evaluation determined |
|  | 5. A priori criteria defined for accuracy / A priori criteria defined for evaluation |
|  | 6. Criterion standard for evaluation stated and different from personal opinion |
| **Scoring system for methodological quality of quantitative included studies (GQ)** | 1. Did the study address a clearly focused issue? |
|  | 2. Did the authors use an appropriate method to answer their question? |
|  | 3. Was the study population clearly specified and defined? |
|  | 4. Were measures taken to accurately reduce measurement bias? |
|  | 5. Were the study data collected in a way that addressed the research issue? |
|  | 6. Did the authors take sufficient steps to assure the quality of the study data? |
|  | 7. Was the data analysis sufficiently rigorous? |
|  | 8. How complete is the discussion? |
|  | 9. To what extent are the findings generalizable to other international contexts? |
| **Scoring system for methodological quality of qualitative included studies (GQ)** | 1. Were steps taken to increase rigour in the analysis of the data? |
|  | 2. Were the findings of the study grounded in/ supported by the data? |
|  | 3. Please rate the findings of the study in terms of their breadth and depth. |
|  | 4. To what extent does the study privilege the perspectives and experiences of health care professionals and patients/carers that are relevant to comparable health systems |
|  | 5. Overall, what weight would you assign to this study in terms of the reliability/ trustworthiness of its findings? |
|  | 6. What weight would you assign to this study in terms of the usefulness of its findings for this review? |
