# Supplementary material for: Prevalence of Health Misinformation on Social Media: Systematic Review
Source: J Med Internet Res. 2021 Jan 20;23(1):e17187. doi: 10.2196/17187 (PMC7857950; doi:10.2196/17187)
Supplement: Multimedia Appendix 3 [file jmir_v23i1e17187_app3.docx]

**Multimedia Appendix 3.** Summary of quality scores.

| Authors | Year | EQ Score | SQ Score | GQ Score |
| --- | --- | --- | --- | --- |
| Abukaraky et al. | 2018 | 71 | 75 | 94 |
| Ahmed et al. | 2019 | 83 | 75 | 100 |
| Al Khaja et al. | 2018 | NA | 38 | 56 |
| Allem et al. | 2017 | NA | 50 | 94 |
| Allem et al.(b) | 2017 | NA | 63 | 78 |
| Arseniev-Koehler et al. | 2016 | NA | 75 | 100 |
| Basch et al. | 2017 | NA | 50 | 78 |
| Becker et al. | 2016 | NA | 63 | 78 |
| Biggs et al. | 2013 | 67 | 75 | 56 |
| Blankenship et al. | 2018 | 43 | 63 | 94 |
| Bora et al. | 2018 | 100 | 63 | 100 |
| Branley et al. | 2017 | NA | 63 | 100 |
| Briones et al. | 2012 | 83 | 75 | 94 |
| Broniatowski et al. | 2018 | 57 | 75 | 100 |
| Buchanan et al. | 2014 | 83 | 44 | 94 |
| Butler et al. | 2013 | 71 | 75 | 94 |
| Cavazos-Rehg et al. | 2018 | 67 | 63 | 94 |
| Chary et al. | 2017 | 50 | 63 | 100 |
| Chew et al. | 2010 | 50 | 75 | 94 |
| Covolo et al. | 2017 | NA | 38 | 67 |
| Dunn et al. | 2015 | 83 | 81 | 100 |
| Dunn et al. | 2017 | NA | 63 | 94 |
| Ekram et al. | 2018 | 33 | 50 | 83 |
| Erdem et al. | 2018 | 21 | 50 | 94 |
| Faasse et al. | 2016 | NA | 38 | 78 |
| Fullwood et al. | 2016 | NA | 50 | 39 |
| Garg et al. | 2015 | 14 | 63 | 83 |
| Gimenez-Perez et al. | 2018 | 100 | 44 | 94 |
| Goobie et al. | 2019 | 83 | 75 | 94 |
| Guidry et al. | 2017 | NA | 50 | 100 |
| Guidry et al. | 2016 | 100 | 75 | 94 |
| Guidry et al. | 2015 | 67 | 75 | 94 |
| Hanson et al. | 2013 | 100 | 63 | 100 |
| Harris et al. | 2018 | 100 | 63 | 78 |
| Haymes et al. | 2016 | 100 | 75 | 94 |
| Helmi et al. | 2018 | 83 | 50 | 94 |
| Kang et al. | 2017 | 43 | 38 | 94 |
| Katsuki et al. | 2015 | 83 | 69 | 100 |
| Keelan et al. | 2010 | 42 | 63 | 100 |
| Keim-Malpass et al. | 2017 | 71 | 63 | 94 |
| Kim et al. | 2017 | 50 | 81 | 94 |
| Krauss et al. | 2017 | 17 | 50 | 94 |
| Krauss et al. | 2015 | 17 | 63 | 94 |
| Kumar et al. | 2014 | 100 | 69 | 94 |
| Laestadius et al. | 2016 | 58 | 75 | 50 |
| Leong et al. | 2018 | 17 | 69 | 94 |
| Lewis et al. | 2015 | NA | 63 | 67 |
| Loeb et al. | 2018 | 33 | 50 | 67 |
| Love et al. | 2013 | 50 | 63 | 94 |
| Martinez et al. | 2018 | 64 | 75 | 94 |
| Massey et al. | 2016 | 36 | 81 | 100 |
| McNeil et al. | 2012 | 33 | 50 | 100 |
| Menon et al. | 2017 | 33 | 63 | 72 |
| Merianos et al. | 2016 | 67 | 75 | 94 |
| Meylakhs et al. | 2014 | NA | 43 | 72 |
| Morin et al. | 2018 | 50 | 75 | 89 |
| Mueller et al. | 2019 | 100 | 63 | 72 |
| Porat et al. | 2019 | 67 | 63 | 78 |
| Radzikowski et al. | 2016 | NA | 88 | 100 |
| Schmidt et al. | 2018 | 57 | 75 | 94 |
| Seltzer et al. | 2017 | 57 | 88 | 94 |
| Seymour et al. | 2015 | 57 | 75 | 94 |
| Syed-Abdul et al. | 2013 | 50 | 75 | 94 |
| Teufel et al. | 2013 | NA | 50 | 94 |
| Tiggermann et al. | 2018 | 14 | 63 | 94 |
| Tuells et al. | 2015 | 83 | 63 | 100 |
| van der Tempel et al. | 2016 | 57 | 75 | 94 |
| Waszak et al. | 2018 | 43 | 63 | 94 |
| Yang et al. | 2018 | 100 | 75 | 94 |
