# Supplementary material for: Prevalence of Health Misinformation on Social Media: Systematic Review
Source: J Med Internet Res. 2021 Jan 20;23(1):e17187. doi: 10.2196/17187 (PMC7857950; doi:10.2196/17187)
Supplement: Multimedia Appendix 4 [file jmir_v23i1e17187_app4.docx]

## Multimedia Appendix 4. Summary table with objectives and conclusions about misinformation prevalence in social media.

| **Authors** | **Year** | **Objectives** | **Methods** | **Topic** | **Social Media Platform** | **Author's conclusions** |
| --- | --- | --- | --- | --- | --- | --- |
| Mueller et al. | 2019 | As little is known about YouTube as a source of information on psoriasis, we aimed to investigate the quality of psoriasis-related videos and, if necessary, point out strategies for their improvement. | Evaluating quality | NCD | YouTube | Two-thirds of the psoriasis-related videos we analyzed disseminate misleading or even dangerous content. |
| Meylakhs et al. | 2014 | We explored three research areas: (1) reasons for newcomers to come to an AIDS-denialist community, (2) the patterns of interactions of the community with the newcomers, and (3) rhetorical strategies that denialists use for persuasion in the veracity of their views. | Social Network Analysis (Netnograpy) | NCD | VK | Contrary to the widespread public health depiction of AIDS denialists as totally irrational, our study suggests that some of those who become AIDS denialists have sufficiently reasonable grounds to suspect that something is wrong with scientific theory, because their personal experience contradicts the unitary picture of AIDS disease progression. |
| Keelan et al. | 2010 | We describe a novel and promising approach to the surveillance of public opinions and attitudes toward immunization. | Content Analysis | Vaccine | MySpace | The high percentage of negative blogs reflected the controversy over the vaccine during its initial adoption. |
| Massey et al. | 2016 | The objectives of our study were to quantify HPV vaccine communication on Twitter, and to develop a novel methodology to improve the collection and analysis of Twitter data. | Sentiment Analysis | Vaccine | Twitter | Using and leveraging social media to detect health trends, as well as communicate important health information, is a growing area of research in public health. |
| Bora et al. | 2018 | We critically evaluated YouTube videos about Zika virus available during the recent Zika pandemic | Evaluating Quality | Pandemic | YouTube | A considerable chunk of the videos were misleading. They were more popular (than informative videos) and could potentially spread misinformation. Videos from trust-worthy sources like university/health organizations were scarce. |
| Kumar et al. | 2014 | We conducted this cross-sectional study to assess the accuracy and content of YouTube videos on HTN and understand how viewers interact with this online information. | Evaluating Quality | NCD | YouTube | Useful videos had the best overall coverage on the epidemiology, pathogenesis, symptoms, complications, preventions/lifestyle modifications, and pharmacologic treatment of HTN. They had a significantly higher quality and reliability score compared with misleading videos and personal views. |
| Gimenez-Perez et al. | 2018 | To evaluate the usefulness of YouTube videos as an educative tool for type 2 diabetes self- management. | Evaluating Quality | NCD | YouTube | Our analysis of YouTube videos as a tool for diabetes self-management education indicates that the probability of finding videos that relate to AADE7 self-care behaviors is less than 50 percent. |
| Buchanan et al. | 2014 | To assess the magnitude, interest, purpose and validity of vaccination-related information on Facebook and to determine whether information varies by site viewpoint. | Sentiment Analysis | Vaccine | Facebook | Facebook, or social media in general, may play a large role in propagation of vaccination misinformation. |
| Dunn et al. | 2015 | We sought to measure whether exposure to negative opinions about human papillomavirus (HPV) vaccines in Twitter communities is associated with the subsequent expression of negative opinions by explicitly measuring potential information exposure over the social structure of Twitter communities. | Social Network Analysis | Vaccine | Twitter | The heterogeneous community structure on Twitter appears to skew the information to which users are exposed in relation to HPV vaccines. |
| Tuells et al. | 2015 | The objective of this work was to know the characteristics of the YouTube videos in Spanish language related to the human papillomavirus vaccine. | Content Analysis | Vaccine | YouTube | Most of the videos have a favorable opinion towards HPV vaccine, although videos with a negative content were the longest and most viewed. |
| Helmi et al. | 2018 | The purpose of this study was to analyze patterns of CWF information dissemination by a network of sources on the web. | Sentiment Analysis | NCD | Different sources | The dominant neutral sentiment of the network may signify that anti- and pro-sides of the debate are viewed as balanced, not just in number but also in quality of information. |
| Teufel et al. | 2013 | To analyze the content and culture of anorexia nervosa (AN)-related communication on the current major social network site (SNS) Facebook. | Evaluating Content | Eating Disorder | Facebook | SNS appears to be a relevant way for young females suffering from AN to communicate and exchange disease and health-related ideas. |
| Faasse et al. | 2016 | Following a prominent Facebook post about childhood vaccination, language used by participants in a comment thread was analysed using LIWC (Linguistic Inquiry and Word Count). | Content Analysis | Vaccine | Facebook | The current findings indicate, that such irrational and emotional qualities do not typify the argument-style or language of Facebook users who make comments indicating opposition to vaccinations. Instead, the antivaccination comments contained linguistic markers of analytical thinking, characterised by categorical language use, often appearing as factual (or in this case, pseudo-factual) and logically structured statements that mimic valid scientific information. |
| Ekram et al. | 2018 | In this observational study we investigated publicly available content regarding the HPV vaccine on the video-sharing Web site YouTube (www.YouTube.com). | Evaluating Content | Vaccine | YouTube | It appears the rhetoric on social media has changed toward mostly anti-vaccine. |
| Porat et al. | 2019 | This study analyses content and source of the most popular tweets related to a recent case in Spain where an unvaccinated child contracted and later died from diphtheria. | Content Analysis | Pandemic | Twitter | The vast majority of popular tweets were either informative or personal opinions expressing frustration or humour/ sarcasm. |
| Menon et al. | 2017 | The purpose of this study is to assess the quality of videos available in YouTube on CyberKnife. | Evaluating Quality | Treatment | YouTube | This study is a mere cross-sectional analysis ofdata available on YouTube on a specific day. It was assessed by three independent oncologists and very definitely subject to physician prejudice against misinformation. However, it was heartening to note that the company videos were reasonably accurate and well presented as were many institutional videos. A totally unexpected benefit from the exercise was the first hand exposure to the profound trust of the patients on the health care system. |
| Meriano et al. | 2016 | This study was conducted to assess the quantity, quality, and reach of e-cigarette health effects YouTube videos, and to quantify the description of positive and negative e-cigarette health effects and promotional content in each video. | Evaluating Content | Drug | YouTube | These unregulated battery-operated products were portrayed as having both negative and beneficial health effects despite inconclusive scientific evidence on the safety of use. For this reason, it is critical to monitor health effects messages on e-cigarettes delivered through YouTube videos and develop appropriate messages to inform consumers about the potential risks associated with product use while mitigating false and misleading information presented. |
| Loeb et al. | 2018 | We performed the largest, most comprehensive examination of prostate cancer information on YouTube to date, including the first 150 videos on screening and treatment. | Evaluating Content | NCD | YouTube | Many popular YouTube videos about prostate cancer contained biased or poor-quality information. A greater number of views and thumbs up on YouTube does not mean that the information is trustworthy. Published |
| Lewis et al. | 2015 | This study examined the nature and scope of NSSI first aid tips on YouTube using a content analysis to examine 40 NSSI first aid videos. | Evaluating Content | Treatment | YouTube | Efforts to provide good quality health information about NSSI via YouTube may be needed. Similar suggestions for other online platforms have also been reported. Mental health professionals also need to be aware that their clients may be accessing videos similar to the ones found in |
| Al Khaja et al. | 2018 | Dissemination of misleading drug information through social media can be detrimental to the health of the public. | Evaluating Quality | Drug | WhatsApp | Majority of the drug- related messages on social media were potentially misleading or false claims that lacked credible evidence to support them. |
| Kim et al. | 2017 | This study aimed to evaluate the accuracy of Korean videos regarding Parkinson’s disease (PD) on YouTube and viewers’ responses to them. | Evaluating Quality | NCD | YouTube | In conclusion, our study found that only about two-thirds of the Korean videos on PD hosted by YouTube provide reliable information. More importantly, the videos with reliable contents were less popular than the videos with misleading information. There were many myths and misconceptions about the etiology and treatment of PD on YouTube, and thus, further efforts are warranted to effectively increase the dissemination of accurate and scientifically proven information on PD to YouTube users. |
| Guidry et al. | 2017 | This study examined Ebola-related social media posts by three major health organizations, Centers for Disease Control and Prevention (CDC), World Health Organization (WHO), Medecins Sans Frontieres (MSF, also known as Doctors without Borders), on Twitter and Instagram, focusing on the types of communication that were used during the outbreak, the content and context of these communications, and the responses they elicited from the publics. | Content Analysis | Pandemic | Twitter Instagram | Overall, less than 3 of Instagram posts and just 1 of tweets addressed Ebola-related misinformation, with no significant differences between the three organizations. Given that misinformation about the disease was especially rampant on social media during the outbreak, these results suggest that health organizations may have missed an important opportunity to highlight and correct misinformation. |
| Chary et al. | 2017 | The purpose of this study was to demonstrate that the geographic variation of social media posts mentioning prescription opioid misuse strongly correlates with government estimates of MUPO in the last month. | Content Analysis | Drug | Twitter | Mentions of MUPO on Twitter correlate strong- ly with state-by-state NSDUH estimates of MUPO. We have also demonstrated that a natural language processing can be used to analyze social media to provide insights for syndromic toxic surveillance. |
| McNeil et al. | 2012 | We sought to explore how seizures are being portrayed on this social networking website and to consider its potential for information dissemination. | Evaluating Content,  Social Network | NCD | Twitter | This study demonstrated the prevalence of stigmatizing |
| Katsuki et al. | 2015 | In order to better assess NUPM behavior online, this study conducts surveillance and analysis of Twitter data to characterize the frequency of NUPM-related tweets and also identifies illegal access to drugs of abuse via online pharmacies. | Evaluating Content, Social Network Analysis | Drug | Twitter | The study also identifies Twitter as a potential source for information illegally promoting the sale of controlled prescription drugs directly to consumers, which is a concerning observation given the inherent risk of abuse, dependency, and questionable authenticity of medicines provided by online pharmacies who are in violation of applicable law, including the US Ryan Haight Act. |
| Becker et al. | 2016 | To gain insight into international public discussion on the paediatric pentavalent vaccine (DTP- HepB-Hib) programme by analysing Twitter messages. | Sentiment Analysis | Vaccine | Twitter | Public messages about DTP-HepB-Hib were characterized by little interaction between tweeters, and by frequent referencing of websites and other information links. Twitter messages can indirectly reflect the public’s opinion about major events in the debates about the DTP-HepB-Hib vaccine. |
| Fullwood et al. | 2016 | Examination of YouTube videos related to synthetic cannabinoids | Evaluating Content | Drug | YouTube | The content of these consumer videos on YouTube often provide the viewer with access to view a wide array of uploaders describing, encouraging, participating and promoting use. |
| Hanson et al. | 2013 | To determine whether people who show signs of prescription drug abuse connect online with others who reinforce this behavior, and to observe the conversation and engagement of these networks with regard to prescription drug abuse. | Evaluating Content, Social Network Analysis | Drug | Twitter | Understanding the prevalence of a problem or issue through social media is a good place to start; however, prevalence data fails to take advantage of the key aspect of social media: social networks and relationships. |
| Krauss et al. | 2017 | To explore the sentiment and themes of Twitter chatter that mentions both alcohol and marijuana. | Evaluating Content | Drug | Twitter | Tweets normalizing polysubstance use or encouraging marijuana use over alcohol use are common. Both online and offline prevention efforts are needed to increase awareness of the risks associated with polysubstance use and marijuana use. Key |
| Garg et al. | 2015 | We assessed the prevalence of the views supporting a link between vaccines and autism online by comparing YouTube, Google and Wikipedia with PubMed | Social Network Analysis | Vaccine | YouTube | Online communities with greater freedom of speech lead to a dominance of anti-vaccine voices |
| Krauss et al. | 2015 | We explored normalization or discouragement of hookah smoking, and other common messages about hookah on Twitter. | Evaluating Content, Social Network Analysis | Drug | Twitter | Educational campaigns about health harms from hookah use and policy changes regarding smoke-free air laws and tobacco advertising on the Internet may be useful to help offset the influence of pro-hookah messages seen on social media |
| Guidry et al. | 2016 | Given the health risks and the misperceptions associated with waterpipe smoking, this study focuses on how waterpipe smoking is portrayed and represented on the social media platform Pinterest | Evaluating Content | Drug | Pinterest | This study focused on Pinterest and concluded that Pinterest portrayals of waterpipe smoking are overwhelmingly positive and almost entirely ignore potential health and addiction risks. |
| Yang et al. | 2018 | The purpose of the study is to investigate how vaping marijuana, a novel but emerging risky health behavior, is portrayed on YouTube, and how the content and features of these YouTube videos influence their popularity and retransmission. | Content Analysis | Drug | YouTube | The results showed that these videos were predominantly pro-marijuana-vaping, with the most frequent videos being user-sharing. The genre and message features influenced the popularity, evaluations, and retransmission of vaping marijuana YouTube videos. |
| Haymes et al. | 2016 | This study aimed to assess the quality of advice contained within YouTube videos on the conservative management of epistaxis. | Evaluating Quality | NCD | YouTube | The quality of information on conservative epistaxis management within YouTube videos is extremely variable. A high search rank is no indication of video quality. Many videos proffer inappropriate and dangerous alternatives advice. We do not recommend YouTube as a source for patient information. |
| Allem et al. | 2017 | This study describes the sentiment of hookah-related posts on Twitter and describes the importance of debiasing Twitter data when attempting to understand attitudes. | Sentiment Analysis | Drug | Twitter | Posts on Twitter communicating positive sentiment toward hookah could add to the normalization of hookah use and is an area of future research. |
| Morin et al. | 2018 | This article presents an analysis of tweets concerning a specific theme: the sexual transmission of the virus by survivors, at a time when there was a great uncertainty about the duration and even the possibility of such transmission. | Sentiment Analysis, Evaluating Content, Social Network Analysis | Pandemic | Twitter | Although numerous studies have shown how this can lead to rumours and disinformation, our research suggest that this relative autonomy makes it possible for Twitter users to bring into the public sphere some types of information that have not been widely addressed. |
| Leong et al. | 2018 | To investigate the content, quality and popularity of information about type 2 diabetes avail- able on YouTube. | Evaluating Quality | NCD | YouTube | The quality of identified videos concerning type 2 diabetes was variable, and misleading videos were popular. Further creation and curation of high-quality video resources is required |
| Dunn et al. | 2017 | Our aim was to determine whether measures of information exposure derived from Twitter could be used to explain differences in coverage in the United States. | Evaluating Content | Vaccine | Twitter | Measures of exposure to HPV related tweets explained more of the variance in state level HPV vaccine coverage than was explained by socioeconomic factors. Our study suggests that in states where negative opinions about HPV vaccines are popularized by mainstream media, the coverage is often lower than would be expected by socioeconomic differences alone. |
| Radzikowski et al. | 2016 | This paper presents a study of Twitter narrative regarding vaccination in the aftermath of the 2015 measles outbreak, both in terms of its cyber and physical characteristics. | Evaluating Content, Social Network Analysis | Vaccine | Twitter | The cyber-physical debate nexus, which connects the cyber narrative in social media to the corresponding geographical space, allows the study of the public’s concerns, views, and responses to health-related issues and thus offers a new avenue for exploring health narratives. As these new mechanisms of discourse are emerging, health communications and health informatics have to adapt to these newfound capabilities and challenges. |
| Harris et al. | 2018 | We sought to 1) examine and compare the characteristics of senders and the content of tweets using these hashtags and 2) identify characteristics associated with engagement with a thinspo or fitspo tweet. | Evaluating Content, Social Network Analysis | Eating Disorder | Twitter | Characteristics of messages and messengers differed between thinspo and fitspo tweets; thinspo tweets were used for messages about disordered eating. Public health professionals should consider using the thinspo hashtag to reach the thinspo group |
| Syed-Abdul et al. | 2013 | The aim of this study was to investigate anorexia-related misinformation disseminated through YouTube videos | Evaluating Content | Eating Disorder | YouTube | Pro-anorexia information was identified in 29.3 of anorexia-related videos. Pro-anorexia videos are less common than informative videos; however, in proportional terms, pro-anorexia content is more highly favored and rated by its viewers. |
| Cavazos-Rehg et al. | 2018 | To investigate tweets about marijuana edibles for surveillance into the content of edibles-related tweets among individuals socially networking about this topic on Twitter | Evaluating Content, Social Network Analysis | Drug | Twitter | Tweets that normalize/encourage edibles use have potential to increase their popularity among individuals who socially network about this topic. Additionally, the prevalence of tweets about edibles’ intense, long-lasting high could have implications for the tailoring of prevention messages that caution potential users against these potential outcomes, which can be important for youth and young adult minorities who were inferred to be disproportionately socially networking about edibles on Twitter. |
| Ahmed et al. | 2019 | Our aim was to utilise an indepth method to study a period of time where the H1N1 Pandemic of 2009 was at its peak | Evaluating Content | Pandemic | Twitter | Misunderstandings of medical advice can lead to dangerous consequences and must be understood carefully |
| Martinez et al. | 2018 | The current study examined conversations on Twitter related to use and perceptions of e-cigarettes in the United States | Evaluating Content | Drug | Twitter | Our findings reveal that although over half of tweets were positive, a sizeable portion was negative or neutral. We also found that, among those tweets mentioning a stigma of e-cigarettes, most confirmed that a stigma does exist. Conversely, among tweets mentioning the harmfulness of e-cigarettes, most denied that e-cigarettes were a health hazard. |
| Guidry et al. | 2015 | This study focused on the social media plat- form Pinterest, analyzing 800 vaccine-related pins through a quantitative content analysis. | Content Analysis | Vaccine | Pinterest | The majority of the pins were anti-vaccine, and most were original posts as opposed to repins. |
| Chew et al. | 2010 | We suggest and evaluate a complementary infoveillance approach using Twitter during the 2009 H1N1 pandemic. | Evaluating Content | Pandemic | Twitter | Content analysis indicated resource-related posts were most commonly shared (52.6). 4.5 of cases were identified as misinformation. |
| Schmidt et al. | 2018 | The goal was to assess whether users’ attitudes are polarized on the topic of vaccination on Facebook and how this polarization develops over time. | Social Network Analysis | Vaccine | Facebook | The existence of echo chambers may explain why social-media campaigns that provide accurate information have limited reach and be effective only in sub-groups, even fomenting further opinion polarization. |
| Branley et al. | 2017 | To compare how people communicate about eating disorders on two popular social media platforms Twitter and Tumblr | Content Analysis | Eating Disorder | Twitter and Tumblr | The results inspire hope that there are positive elements to online communication about ED such as inspiring recovery, raising awareness, and challenging societal norms. However, it is vital to ensure that pro-ana content is not trivialized or dismissed. |
| Arseniev-Koehler et al. | 2016 | The purpose of this study was to investigate Pro-ED Twitter profiles’ references to EDs and how their social connections (followers) reference EDs | Evaluating Content, Social Network Analysis | Eating Disorder | Twitter | Findings suggest that profiles which self-identify as Pro-ED express disordered eating patterns through tweets and have an audience of followers, many of whom also reference ED in their own profiles. ED socialization on Twitter might provide social support, but in the Pro- ED context this activity might also reinforce an ED identity |
| Seltzer et al. | 2017 | We sought to explore how the image-sharing platform Instagram is used for information dissemination and conversation during the current Zika outbreak | Evaluating Content | Pandemic | Instagram | These insights are useful in assessing fears and public opinion that could allow for more targeted surveillance, education, and intervention. As more individuals are affected and the conversation surrounding Zika evolves it will be important to provide salient information in forums where individuals are already frequent, including social media based image platforms. |
| Butler et al. | 2013 | To evaluate the clinical accuracy and delivery of information on thermal burn first aid available on the leading video-streaming website, YouTube | Evaluating Quality | Treatment | YouTube | The current standard of videos covering thermal burn first aid available on YouTube is unsatisfactory. In addition to this, viewers do not appear to be drawn to videos of higher quality |
| Abukaraky et al. | 2018 | To examine what YouTube offers patients seeking information on dental implants, and to evaluate the quality of provided information | Evaluating Quality | Treatment | YouTube | Information about dental implants on YouTube is limited in quality and quantity. |
| Erdem et al. | 2018 | The aim of this study was to answer the question: Is watching these videos useful to surgeons and patients? | Evaluating Quality | Treatment | YouTube | No misleading information was found |
| Kang et al. | 2017 | To examine current vaccine sentiment on social media by constructing and analyzing semantic networks of vaccine information from highly shared websites of Twitter users in the United States; and to assist public health communication of vaccines | Content Analysis | Vaccine | Twitter | Semantic network analysis of vaccine sentiment in online social media can enhance understanding of the scope and variability of current attitudes and beliefs toward vaccines. Our study synthesizes quantitative and qualitative evidence from an interdisciplinary approach to better understand complex drivers of vaccine hesitancy for public health communication, to improve vaccine confidence and vaccination coverage in the United State. |
| Blankenship et al. | 2018 | To investigate if tweets with different sentiments toward vaccination and different contents attract different levels of Twitter users’ engagement. | Social Network Analysis | Vaccine | Twitter | Engaging social media key opinion leaders to facilitate health education about vaccination in their tweets may allow reaching a wider audience online |
| Waszak et al. | 2018 | Our pilot study is an initial attempt to measure a number of the top shared health misinformation stories in the Polish language social media. | Evaluating Content | NCD | Facebook | Analyzing social media top shared news could contribute to identification of leading fake medical information miseducating the society. It might also encourage authorities to take actions such as put warnings on biased domains or scientifically evaluate those generating fake health news |
| Tiggermann et al. | 2018 | The aim of the present study was to compare thinspiration and fitspiration communities on Twitter. | Social Network Analysis | Eating Disorder | Twitter | Frequency counts and sentiment analysis showed that although the tweets from both types of accounts focused on appearance and weight loss, fitspiration tweets were significantly more positive in sentiment. It was concluded that the thinspiration tweeters, unlike the fitspiration tweeters, represent a genuine on-line community on Twitter. Such a community of support may have negative consequences for collective body image and disordered eating identity |
| Love et al. | 2013 | This study reports a content analysis of posts about vaccinations, documenting sources, tone, and medical accuracy | Social Network Analysis | Vaccine | Twitter | Clinicians must be prepared to address patients entering the clinical environment with opinions and expectations based on social media sources and shared links, possibly including false impressions about adverse effects or unsupported expectations for vaccine effectiveness |
| Keim-Malpass et al. | 2017 | The purpose of this study was to evaluate the content of messaging regarding the HPV vaccine on the social media and microblogging site Twitter, and describe the sentiment of those messages. | Evaluating Content | Vaccine | Twitter | Using Twitter to understand public sentiment offers a novel perspective to explore the context of health communication surrounding certain controversial issues |
| van der Tempel et al. | 2016 | Individuals seeking information about electronic cigarettes are increasingly turning to social media networks like Twitter. We surveyed dominant Twitter communications about e-cigarettes and smoking cessation, examining message sources, themes, and attitudes. | Social Network Analysis | Drug | Twitter | Our findings show that Twitter users are overwhelmingly exposed to messages that favor e-cigarettes as smoking cessation aids, even when disregarding commercial activity. This underlines the need for effective public health engagement with social media to provide reliable information about e-cigarettes and smoking cessation online |
| Laestadius et al. | 2016 | This exploratory study analyzed electronic cigarette content found on the visual social networking service, Instagram, in order to highlight public health challenges created by this content and support understanding of electronic cigarette promotion and usage. | Evaluating Content | Drug | Instagram | Instagram content related to e-cigarettes poses two primary areas for concern: (1) e-cigarette users, brands, and vendors are exposing their followers to e-cigarette content, (2) users themselves may reinforce their identity and community membership as vaper through their creation of content and hashtags. |
| Broniatowski et al. | 2018 | To understand how Twitter bots and trolls promote online health. | Content Analysis | Vaccine | Twitter | Accounts unlikely to be bots are significantly less likely to promote polarized and antivaccine content. Nevertheless, bots and trolls are actively involved in the online public health discourse, skewing discussions about vaccination. |
| Covolo et al. | 2017 | The aim of this study was to explore the message available on YouTube videos about vaccination. | Evaluating Content, Social Network Analysis | Vaccine | YouTube | Considering the increasing use of social media, it would be worth to further investigate how this tool can be used to promote vaccination. It should be also considered that young people are shown to be more sensitive to immunization promotion messages received through social media |
| Basch et al. | 2017 | Using the keywords “vaccine safety” and “vaccines and children”, 87 of the most widely viewed YouTube videos were identified and analyzed for content, author status and view count. | Evaluating Content | Vaccine | YouTube | Health professionals should be aware of the widely disseminated vaccination information available on the Internet and should appreciate its possible effect on the public. |
| Seymour et al. | 2015 | In an antifluoridation case study, we explored digital pandemics and the social spread of scientifically inaccurate health information across the Web, and we considered the potential health effects | Social Network Analysis | NCD | Facebook | Network sociology may be as influential as the information content and scientific validity of a particular health topic discussed using social media. Public health must employ social strategies for improved communication management. |
| Briones et al. | 2012 | This article reports a content analysis of YouTube videos related to the human papillomavirus (HPV) vaccine. | Evaluating Content, Social Network Analysis | Vaccine | YouTube | In conclusion, the results from this study demonstrate that the tone has somewhat shifted on YouTube in terms of the HPV vaccine. Even though most videos were positive only a couple of years ago, more users have since posted content that is more critical of the vaccine. These findings show that YouTube has the potential to shift attitudes and beliefs about a controversial topic such as the HPV vaccine in a relatively short period of time. |
| Biggs et al. | 2013 | This study aimed to determine whether YouTube represented a valid and reliable patient information resource for the lay person on the topic of rhinosinusitis. | Evaluating Quality | NCD | YouTube | YouTube appears to be an unreliable resource for accurate and up to date medical information relating to rhinosinusitis. However, it may provide some useful information if mechanisms existed to direct lay people to verifiable and credible sources. |
| Goobie et al. | 2019 | We aimed to determine viewer engagement, quality, and content of YouTube videos on IPF and to compare the provided information with contemporaneous guidelines. | Evaluating Quality | NCD | YouTube | Patient-directed YouTube videos on IPF frequently provide incomplete and inaccurate information. Videos supporting the use of non-recommended therapies have higher viewing numbers and user engagement, highlighting the potential risks of using YouTube as a resource for health information. |
| Allem et al.(b) | 2017 | This study documents e-cigarette–related discussions on Twitter, describing themes of conversations and locations where Twitter users often discuss e-cigarettes, to identify priority areas for e-cigarette education campaigns. Additionally, this study demonstrates the importance of distinguishing between social bots and human users when attempting to understand public health–related behaviors and attitudes. |  | Drug | Twitter | Social media data may be used to complement and extend the surveillance of health behaviors including tobacco product use. Social bots may be used to perpetuate the idea that e-cigarettes are helpful in cessation and to promote new products as they enter the marketplace. |
